# Subtype Characterization and Zoonotic Potential of *Cryptosporidium felis* in Cats in Guangdong and Shanghai, China

**DOI:** 10.3390/pathogens10020089

**Published:** 2021-01-20

**Authors:** Jiayu Li, Fuxian Yang, Ruobing Liang, Sheng Guo, Yaqiong Guo, Na Li, Yaoyu Feng, Lihua Xiao

**Affiliations:** 1 Center for Emerging and Zoonotic Diseases, College of Veterinary Medicine, South China Agricultural University, Wushan Road, Guangzhou 510642, China; hnnydxsyljy@126.com (J.L.); FuxianYang1210@163.com (F.Y.); tuzi0411@126.com (R.L.); guosheng201911@163.com (S.G.); guoyq@scau.edu.cn (Y.G.); nli@scau.edu.cn (N.L.); 2 Guangdong Laboratory for Lingnan Modern Agriculture, Wushan Road, Guangzhou 510642, China

**Keywords:** *Cryptosporidium felis*, 60-kDa glycoprotein, subtypes, zoonotic transmission

## Abstract

*Cryptosporidiumfelis* is an important cause of feline and human cryptosporidiosis. However, the transmission of this pathogen between humans and cats remains controversial, partially due to a lack of genetic characterization of isolates from cats. The present study was conducted to examine the genetic diversity of *C. felis* in cats in China and to assess their potential zoonotic transmission. A newly developed subtyping tool based on a sequence analysis of the 60-kDa glycoprotein (*gp60*) gene was employed to identify the subtypes of 30 cat-derived *C. felis* isolates from Guangdong and Shanghai. Altogether, 20 *C. felis* isolates were successfully subtyped. The results of the sequence alignment showed a high genetic diversity, with 13 novel subtypes and 2 known subtypes of the XIXa subtype family being identified. The known subtypes were previously detected in humans, while some of the subtypes formed well-supported subclusters with human-derived subtypes from other countries in a phylogenetic analysis of the *gp60* sequences. The results of this study confirmed the high genetic diversity of the XIXa subtype family of *C. felis*. The common occurrence of this subtype family in both humans and cats suggests that there could be cross-species transmission of *C. felis*.

## 1. Introduction

*Cryptosporidium* spp. are important apicomplexan parasites inhabiting the gastrointestinal tract of humans and other vertebrates, causing severe diarrhea [1]. Human cryptosporidiosis has been associated with over 20 *Cryptosporidium* species, but *C. hominis*, *C. parvum*, *C. meleagridis*, *C. felis*, and *C. canis* are the most common ones [2]. Among them, *C. felis* mainly infects cats and is therefore considered a host-adapted species [3]. Human *C. felis* infections, however, are common in developing countries [4,5,6,7], and at least one possible zoonotic transmission of *C. felis* between a household cat and the owner has been reported [8]. Nevertheless, the limited number of reports of zoonotic infections with this species has raised questions on the importance of zoonotic transmission in the epidemiology of human *C. felis* infections [9].

Subtyping tools based on sequence analysis of the 60-kDa glycoprotein (*gp60*) gene have been developed for human-pathogenic *Cryptosporidium* spp. to track infection sources [10]. Currently, the *gp60*-based subtyping tools are available for *C. hominis*, *C. parvum*, *C. meleagridis*, *C. ubiquitum*, *C. viatorum*, *Cryptosporidium* skunk genotype, and *Cryptosporidium* chipmunk genotype I [11,12,13,14,15,16]. These subtyping methods have been used in characterizing the transmission of these *Cryptosporidium* spp. in humans and animals [3].

A *gp60* subtyping tool has been developed recently for genetic characterizations of *C. felis* [17]. Thus far, nearly 200 *C. felis* isolates have been examined, which has led to the identification of approximately 100 subtypes in five subtype families (XIXa, XIXb, XIXc, XIXd, and XIXe) worldwide [17,18]. Most of the isolates, however, were from humans, and only two isolates from a human and a rhesus macaque have been characterized from China [18]. As *C. felis* has been identified in children and immunocompromised patients in China [19,20,21,22], we sought to examine the subtype identity of cat-derived *C. felis* isolates from Guangdong and Shanghai for assessment of their zoonotic potential.

## 2. Results

### 2.1. Amplification of the gp60 Gene

**Figure 1:**
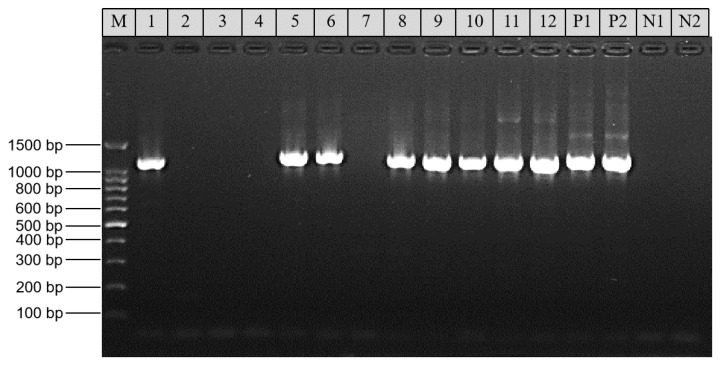
Analysis of the 60-kDa glycoprotein (*gp60*) gene in *Cryptosporidium felis* by nested PCR: lanes 1 and 2, SCAU320; lanes 3 and 4, SCAU2396; lanes 5 and 6, SCAU1149; lanes 7 and 8, SCAU1850; lanes 9 and 10, SCAU1851; lanes 11 and 12, SCAU1857; lane M, 100-bp molecular marker; P1 and P2, positive control (*C. felis* DNA); and N1 and N2, negative control (reagent-grade water).

Among the 30 DNA preparations that were positive for *C. felis* based on nested PCR analysis of the small subunit (SSU) rRNA gene, 20 (66.7%) generated the expected products in the *gp60* PCR. Among them, 9 were from cats in pet shelters, 5 were from cats visiting animal hospitals, 4 were from cats in pet shops, and 2 were from stray cats (Table 1). These PCR products differed slightly in size (Figure 1) and were all sequenced successfully.

### 2.2. Nucleotide Sequence Variations in the gp60 Gene of C. felis

A comparison of the nucleotide sequences generated led to the identification of 15 sequence types. They differed from each other by both nucleotide substitutions and copy numbers of repetitive sequences. The sequence alignments showed the presence of numerous single nucleotide substitutions (SNPs) over the partial *gp60* gene among the 20 *C. felis* isolates. Three types of simple tandem repeats were detected in the *gp60* genes (Table 2). Among them, 2–5 copies of the 33-bp repeat sequence 5′-CCACCTAGTGGCGGTAGTGGCGTGTCCCCTGCT-3′ and 2–4 copies of the 39-bp repeat sequence 5′-CCACCTAGTGGCGGTAGTGGCGTGTCCCCTGCT-3′ were observed at nucleotides 460–577 and 778–911 of the sequence alignment, respectively. In both repeat types, the last copy had only half of the usual length. In addition, 5 copies of the trinucleotide repeat GTT were found in the *gp60* sequences at nucleotides 1143-1154.

### 2.3. Cryptosporidium felis Subtypes Identified

Altogether, 15 subtypes were identified, with two subtypes in samples SCAU392 and SCAU754 having nucleotide sequences identical to the XIXa-39 and XIXa-40 subtypes (GenBank reference sequences MH240852 and MH240853) from humans in the United Kingdom, respectively (Table 1). According to the nomenclature system of the XIXa subtypes [18], the 13 new subtypes were named XIXa-81 (3), XIXa-82 (1), XIXa-83 (1), XIXa-84 (1), XIXa-85 (1), XIXa-86 (1), XIXa-87 (2), XIXa-88 (2), XIXa-89 (2), XIXa-90 (1), XIXa-91 (1), XIXa-92 (1), and XIXa-93 (1).

### 2.4. Phylogenetic Analysis of C. felis Subtypes

**Figure 2:**
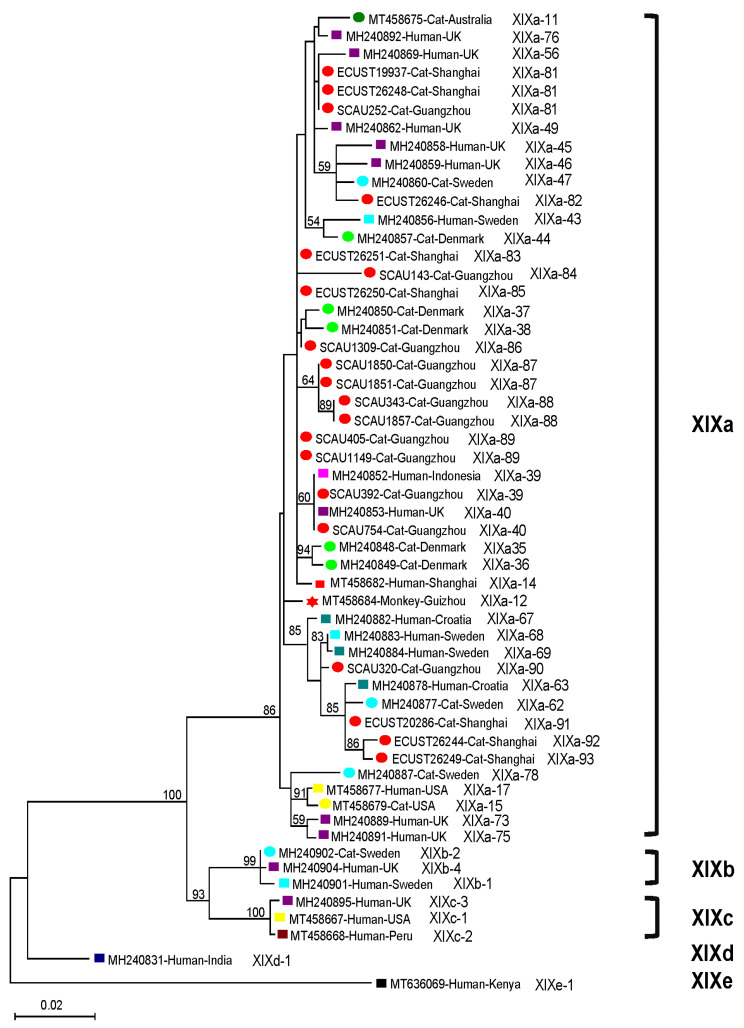
Phylogenetic relationship among XIXa subtypes of *Cryptosporidium felis* identified in this study and references from GenBank based on maximum likelihood analysis of the sequences of partial *gp60* gene: bootstrap values over 50 percent are shown on the branches of the phylogenetic tree. The human-, monkey-, and cat-derived isolates are indicated by rhombus, star, and round labels, respectively. Sequences from different countries are shown in different colors. The names of known subtype families of *C. felis* are shown on the right side of the phylogenetic tree. The subtype name of each isolate is labeled at the end of the sequence.

The *gp60* sequences from the 20 *C. felis* isolates were all placed in the XIXa subtype family in the phylogenetic tree (Figure 2). They formed several subclusters with strong bootstrap support with *C. felis* subtypes from humans. Among the novel subtypes identified in the study, 4 subtypes (XIXa-90, XIXa-91, XIXa-92, and XIXa-93) formed a subcluster with several subtypes identified from humans and cats in several European countries and another subtype (XIXa-82) formed a subcluster with several subtypes from humans and cats in the United Kingdom and Sweden. In addition, two cat-derived subtypes (XIXa-87 and XIXa-88) in 4 isolates from this study formed their own subcluster. As expected, the known subtypes (XIXa-39 and XIXa-40) identified in the present study and previously in humans from the United Kingdom and Indonesia formed a subcluster (Figure 2).

## 3. Discussion

The *gp60* subtyping tools have been widely used in assessing the intra-species diversity and zoonotic transmission of human-pathogenic *Cryptosporidium* spp. Several genes including *gp60*, other genetic loci with simple tandem repeats, and double-stranded viral RNA have been used to further differentiate the *Cryptosporidium* species into different subtypes [10]. Among them, the *gp60* gene is highly polymorphic in all *Cryptosporidium* spp. examined thus far and therefore can be categorized into multiple subtype families by nucleotide sequence differences [4]. Moreover, the *gp60* is an invasion-related protein and the subtype families identified have been linked to differences in host ranges and with virulence in *C. parvum* and *C. hominis* [3,23,24]. In this study, the newly developed *gp60* subtyping tool was employed to characterize the *C. felis* and to understand its zoonotic potential in China.

This represents the first subtyping study of *C. felis* in China. The previous two studies on *C. felis* subtypes mostly examined *C. felis* isolates from other countries, with five subtype families (XIXa, XIXb, XIXc, XIXd, and XIXe) being identified [17,18]. In the present study, 20 cat-derived isolates from China were subtyped and the results of the phylogenetic analysis showed that all of them belonged to the subtype family XIXa. The PCR amplification efficiency (66.7%) of this study was similar to that of a previous study (67.0%) [18]. The light infections of *C. felis* in healthy cats could be one of the reasons for the relatively low PCR amplification efficiency.

An analysis of the *gp60* gene confirmed the high genetic diversity of *C. felis* isolates. Like observations in previous studies [17,18], 15 subtypes were seen in 20 *C. felis* isolates successfully analyzed from cats. However, all *C. felis* isolates in this study belonged to the subtype family XIXa. As 13 of the 15 XIXa subtypes identified were novel, there could be geographic isolation among some of the XIXa subtypes, as previously suggested by us [18].

The results of the present study suggest that cross-species transmission of *C. felis* could be possible. In this study, among the 15 XIXa subtypes, two were identical to subtypes previously found in humans, while others clustered with human-derived subtypes from other countries. Importantly, one of the subclusters formed included two sequences from an owner and the household cat (GenBank reference sequences MH240883 and MH240884) with possible zoonotic transmission [17]. Moreover, we previously subtyped two *C. felis* isolates from one child in Shanghai (XIXa-14) and one rhesus macaque (XIXa-12) in Guizhou, China [18], while the *gp60* sequences were different with the 15 XIXa subtypes identified in the present study. They clustered together in a large clade within the XIXa subtype family in the phylogenetic tree (Figure 2). The subtype characteristics of *C. felis* identified in this suggest that the zoonotic XIXa subtype family could be the dominant subtypes in pet cats in China. As the sequences from *C. felis* in Guangzhou and Shanghai are dispersed throughout the large clade within the XIXa subtype family, they could be good representatives of *C. felis* in cats in China. Since *C. felis* infections have been reported in cats and humans in several locations in China [19,25,26,27,28], an analysis of the *gp60* sequences of human- and cat-derived *C. felis* isolates from the same location is needed to understand the epidemiological importance of zoonotic transmission of *C. felis*.

Currently, the significance of the divergent *C. felis* subtypes in cats is not clear. As mentioned above, different *C. parvum* and *C. hominis* subtypes have been linked to differences in virulence. Therefore, the hyper-transmissible and virulent *C. hominis* IbA10G2 and *C. parvum* IIaA15G2R1 subtypes have caused numerous outbreaks of human cryptosporidiosis worldwide [10] and infections with the former have induced more clinical symptoms than other *C. hominis* subtypes [23]. In *C. felis*, although previous studies and results of the present study suggest that the XIXa is the dominant subtype family in humans and cats [18], the relationship between the pathogenicity and its high transmission is not yet clear. More subtyping studies at additional genetic loci are needed to understand the differences in pathogenicity among *C. felis* subtypes.

## 4. Materials and Methods

### 4.1. Ethics Statement

This research was reviewed and approved by the Ethics Committee of the South China Agricultural University. The fecal specimens were collected with the permission of the owners of the pets. Each sample was collected and placed into a 50-mL plastic centrifuge tube containing 2.5% potassium dichromate and was transferred to the laboratory for storage at 4 °C. The DNA extraction was completed within one week. The DNA was stored at −20 °C for less than three years before being used in the PCR analysis in the present study.

### 4.2. C. felis Isolates

The nested PCR targeting the small subunit (SSU) rRNA gene was used to detect *Cryptosporidium* spp. [29]. DNA preparations of 30 *C. felis*-positive fecal samples from China were used in the present study. Among them, 18 samples were obtained from cats in Guangdong province, including 7 from pet shelters, 6 from animal hospitals, 4 from stray cats, and 1 from a pet shop. The remaining 12 samples were obtained from cats in Shanghai, including 8 from pet shelters and 4 from pet shops. They were from two previous studies of molecular epidemiology of cryptosporidiosis in cats [27,28].

### 4.3. PCR Amplification

The newly developed nested PCR targeting the conserved region of the *gp60* gene was employed to identify the subtypes of *C. felis* in this study [17]. Briefly, primers GP60-Felis-F1 (5′-TTT CCG TTA TTG TTG CAG TTG CA-3′) and GP60-Felis-R1 (5′-ATC GGA ATC CCA CCA TCG AAC-3′) were used in primary PCR, while GP60-Felis-F2 (5′-GGG CGT TCT GAA GGA TGT AA-3′) and GP60-Felis-R2 (5′-CGG TGG TCT CCT CAG TCT TC-3′) were used in secondary PCR. The sizes of primary and secondary PCR products were approximately 1200 and 900 bp, respectively. The PCR reaction and cycling program were described recently [18]. Each DNA preparation was analyzed in duplicate, with the inclusion of both positive (*C. felis* DNA) and negative (reagent-grade water) controls in each PCR run. The positive products from the secondary PCR were identified by 1.5% agarose electrophoresis.

### 4.4. DNA Sequence Analyses

All positive products of the expected size were sequenced bidirectionally on an ABI3730 autosequencer by the Sangon Biotech (Shanghai, China) using secondary PCR primers. The DNA sequences generated were assembled using ChromasPro 2.1.6 (http://technelysium.com.au/wp/) and edited using BioEdit 7.1.3.0 (http://www.mbio.ncsu.edu/BioEdit/bioedit.html). These and the reference sequences from GenBank were aligned with each other using the MUSCLE program implemented in MEGA 6 (https://www.megasoftware.net/). Tandem Repeats Finder 4.09 (http://tandem.bu.edu/trf/trf.html) was used to identify repetitive sequences within them. A maximum-likelihood tree was constructed using MEGA 6 based on substitution rates calculated using the general time reversible model and gamma distribution. The bootstrap method with 1000 replicates was used to assess the reliability of the phylogenetic clusters formed. Representative nucleotide sequences of the *C. felis* subtypes identified in the present study were deposited in the GenBank database under accession numbers MW351820-MW351832.

## 5. Conclusions

The present study reported the subtype characteristics of *C. felis* isolates from cats in China for the first time. The results of the phylogenetic analysis suggested the potential zoonotic transmission of this pathogen. More isolates from diverse areas and hosts should be analyzed to confirm this conclusion.

## Figures and Tables

**Table 1:** Sources of *Cryptosporidium felis* samples used in the study and their *gp60* subtype identity.

Sample ID	Host	Region	Sample Source	Subtype
SCAU320	Cat	Guangzhou	Animal hospital	XIXa-90
SCAU1149	Cat	Guangzhou	Animal hospital	XIXa-89
SCAU1850	Cat	Guangzhou	Animal hospital	XIXa-87
SCAU1851	Cat	Guangzhou	Animal hospital	XIXa-87
SCAU1857	Cat	Guangzhou	Animal hospital	XIXa-88
SCAU2396	Cat	Shenzhen	Animal hospital	N/A
SCAU252	Cat	Guangzhou	Pet shop	XIXa-81
SCAU343	Cat	Guangzhou	Pet shelter	XIXa-88
SCAU356	Cat	Guangzhou	Pet shelter	N/A
SCAU392	Cat	Guangzhou	Pet shelter	XIXa-39
SCAU405	Cat	Guangzhou	Pet shelter	XIXa-89
SCAU1309	Cat	Shantou	Pet shelter	XIXa-86
SCAU4854	Cat	Guangzhou	Pet shelter	N/A
SCAU4905	Cat	Guangzhou	Pet shelter	N/A
SCAU143	Cat	Guangzhou	Stray animal	XIXa-84
SCAU731	Cat	Guangzhou	Stray animal	N/A
SCAU732	Cat	Guangzhou	Stray animal	N/A
SCAU754	Cat	Guangzhou	Stray animal	XIXa-40
ECUST19937	Cat	Shanghai	Pet shop	XIXa-81
ECUST26245	Cat	Shanghai	Pet shop	N/A
ECUST26246	Cat	Shanghai	Pet shop	XIXa-82
ECUST26248	Cat	Shanghai	Pet shop	XIXa-81
ECUST19997	Cat	Shanghai	Pet shelter	N/A
ECUST20283	Cat	Shanghai	Pet shelter	N/A
ECUST20286	Cat	Shanghai	Pet shelter	XIXa-91
ECUST20309	Cat	Shanghai	Pet shelter	N/A
ECUST26244	Cat	Shanghai	Pet shelter	XIXa-92
ECUST26249	Cat	Shanghai	Pet shelter	XIXa-93
ECUST26250	Cat	Shanghai	Pet shelter	XIXa-85
ECUST26251	Cat	Shanghai	Pet shelter	XIXa-83

N/A: PCR negative.

**Table 2:** Tandem repeats in nucleotide sequences of the *gp60* gene of *Cryptosporidium felis.*

Sample ID ^a^	GenBank Accession no.	Subtype	33-bp Repeat (No.) at 460–577 bp	39-bp Repeat (No.) at 778–911 bp	GGT Repeat (No.) at 1143–1154
SCAU392	MH240852	XIXa-39	R1 ^b^ (2)	R2 ^c^ (3)	4
SCAU754	MH240853	XIXa-40	R1 (3)	R2 (3)	4
SCAU252	MW351820	XIXa-81	R1 (2)	R2 (4)	4
ECUST19937	MW351820	XIXa-81	R1 (2)	R2 (4)	4
ECUST26248	MW351820	XIXa-81	R1 (2)	R2 (4)	4
ECUST26246	MW351821	XIXa-82	-	R2 (2)	4
ECUST26251	MW351822	XIXa-83	-	R2 (3)	4
SCAU143	MW351823	XIXa-84	R1 (5)	R2 (3)	4
ECUST26250	MW351824	XIXa-85	-	R2 (3)	4
SCAU1309	MW351825	XIXa-86	-	R2 (3)	4
SCAU1850	MW351826	XIXa-87	R1 (2)	R2 (2)	4
SCAU1851	MW351826	XIXa-87	R1 (2)	R2 (2)	4
SCAU343	MW351827	XIXa-88	R1 (2)	R2 (2)	4
SCAU1857	MW351827	XIXa-88	R1 (2)	R2 (2)	4
SCAU405	MW351828	XIXa-89	R1 (2)	R2 (3)	4
SCAU1149	MW351828	XIXa-89	R1 (2)	R2 (3)	4
SCAU320	MW351829	XIXa-90	R1 (2)	R2 (4)	4
ECUST20286	MW351830	XIXa-91	R1 (2)	R2 (3)	4
ECUST26244	MW351831	XIXa-92	R1 (2)	R2 (3)	4
ECUST26249	MW351832	XIXa-93	R1 (2)	R2 (3)	4

^a^ Sample IDs labelled with ECUST were from cats in Shanghai, while those with SCAU were from cats in Guangdong; ^b^ 33-bp tandem repeat (5′-CCACCTAGTGGCGGTAGTGGCGTGTCCCCTGCT-3′) with a partial copy at the end; ^c^ 39-bp tandem repeat (5′-AGCACAACTGCGGCTACAGCGAGCACTGCGAGTTCGACA-3′) with a partial copy at the end and 0–2 nucleotide differences.

## Data Availability

Data is contained within the article.

## References

[1] Checkley W., White A.C., Jaganath D., Arrowood M.J., Chalmers R.M., Chen X.M., Fayer R., Griffiths J.K., Guerrant R.L., Hedstrom L. (2015). A review of the global burden, novel diagnostics, therapeutics, and vaccine targets for *Cryptosporidium*. Lancet Infect. Dis..

[2] Zahedi A., Ryan U. (2020). *Cryptosporidium*—An update with an emphasis on foodborne and waterborne transmission. Res. Vet. Sci..

[3] Feng Y., Ryan U.M., Xiao L. (2018). Genetic diversity and population structure of *Cryptosporidium*. Trends Parasitol..

[4] Xiao L. (2010). Molecular epidemiology of cryptosporidiosis: An update. Exp. Parasitol..

[5] de Lucio A., Merino F.J., Martinez-Ruiz R., Bailo B., Aguilera M., Fuentes I., Carmena D. (2016). Molecular genotyping and sub-genotyping of *Cryptosporidium* spp. isolates from symptomatic individuals attending two major public hospitals in Madrid, Spain. Infect. Genet. Evol..

[6] Cieloszyk J., Goni P., Garcia A., Remacha M.A., Sanchez E., Clavel A. (2012). Two cases of zoonotic cryptosporidiosis in Spain by the unusual species *Cryptosporidium ubiquitum* and *Cryptosporidium felis*. Enferm. Infecc. Microbiol. Clin..

[7] Caccio S., Pinter E., Fantini R., Mezzaroma I., Pozio E. (2002). Human infection with *Cryptosporidium felis*: Case report and literature review. Emerg. Infect. Dis..

[8] Beser J., Toresson L., Eitrem R., Troell K., Winiecka-Krusnell J., Lebbad M. (2015). Possible zoonotic transmission of *Cryptosporidium felis* in a household. Infect. Ecol. Epidemiol..

[9] Lucio-Forster A., Griffiths J.K., Cama V.A., Xiao L., Bowman D.D. (2010). Minimal zoonotic risk of cryptosporidiosis from pet dogs and cats. Trends Parasitol..

[10] Xiao L., Feng Y. (2017). Molecular epidemiologic tools for waterborne pathogens *Cryptosporidium* spp. and *Giardia duodenalis*. Food Waterborne Parasitol..

[11] Yan W., Alderisio K., Roellig D.M., Elwin K., Chalmers R.M., Yang F., Wang Y., Feng Y., Xiao L. (2017). Subtype analysis of zoonotic pathogen *Cryptosporidium* skunk genotype. Infect. Genet. Evol..

[12] Stensvold C.R., Elwin K., Winiecka-Krusnell J., Chalmers R.M., Xiao L., Lebbad M. (2015). Development and application of a gp60-based typing assay for *Cryptosporidium viatorum*. J. Clin. Microbiol..

[13] Guo Y., Cebelinski E., Matusevich C., Alderisio K.A., Lebbad M., McEvoy J., Roellig D.M., Yang C., Feng Y., Xiao L. (2015). Subtyping novel zoonotic pathogen *Cryptosporidium* chipmunk genotype I. J. Clin. Microbiol..

[14] Stensvold C.R., Beser J., Axen C., Lebbad M. (2014). High applicability of a novel method for gp60-based subtyping of *Cryptosporidium meleagridis*. J. Clin. Microbiol..

[15] Li N., Xiao L., Alderisio K., Elwin K., Cebelinski E., Chalmers R., Santin M., Fayer R., Kvac M., Ryan U. (2014). Subtyping *Cryptosporidium ubiquitum*, a zoonotic pathogen emerging in humans. Emerg. Infect. Dis..

[16] Sulaiman I.M., Hira P.R., Zhou L., Al-Ali F.M., Al-Shelahi F.A., Shweiki H.M., Iqbal J., Khalid N., Xiao L. (2005). Unique endemicity of cryptosporidiosis in children in Kuwait. J. Clin. Microbiol..

[17] Rojas-Lopez L., Elwin K., Chalmers R.M., Enemark H.L., Beser J., Troell K. (2020). Development of a gp60-subtyping method for *Cryptosporidium felis*. Parasites Vectors.

[18] Jiang W., Roellig D.M., Lebbad M., Beser J., Troell K., Guo Y., Li N., Xiao L., Feng Y. (2020). Subtype distribution of zoonotic pathogen *Cryptosporidium felis* in humans and animals in several countries. Emerg. Microbes Infect..

[19] Feng Y., Wang L., Duan L., Gomez-Puerta L.A., Zhang L., Zhao X., Hu J., Zhang N., Xiao L. (2012). Extended outbreak of cryptosporidiosis in a pediatric hospital, China. Emerg. Infect. Dis..

[20] Chen S., Ai L., Tian L., Zhang Y., Tong X., Li H., Chen J. (2012). Investigation and fecal specimens detection of cryptozoite and other protozoon infection from patients with diarrhea. Chin. J. Zoonoses.

[21] Hung C.C., Tsaihong J.C., Lee Y.T., Deng H.Y., Hsiao W.H., Chang S.Y., Chang S.C., Su K.E. (2007). Prevalence of intestinal infection due to *Cryptosporidium* species among Taiwanese patients with human immunodeficiency virus infection. J. Formos. Med. Assoc..

[22] Wang R., Zhang X., Zhu H., Zhang L., Feng Y., Jian F., Ning C., Qi M., Zhou Y., Fu K. (2011). Genetic characterizations of *Cryptosporidium* spp. and *Giardia duodenalis* in humans in Henan, China. Exp. Parasitol..

[23] Cama V.A., Bern C., Roberts J., Cabrera L., Sterling C.R., Ortega Y., Gilman R.H., Xiao L. (2008). *Cryptosporidium* species and subtypes and clinical manifestations in children, Peru. Emerg. Infect. Dis..

[24] Cama V.A., Ross J.M., Crawford S., Kawai V., Chavez-Valdez R., Vargas D., Vivar A., Ticona E., Navincopa M., Williamson J. (2007). Differences in clinical manifestations among *Cryptosporidium* species and subtypes in HIV-infected persons. J. Infect. Dis..

[25] Liu A., Gong B., Liu X., Shen Y., Wu Y., Zhang W., Cao J. (2020). A retrospective epidemiological analysis of human *Cryptosporidium* infection in China during the past three decades (1987–2018). PLoS Negl. Trop. Dis..

[26] Li W., Liu X., Gu Y., Liu J., Luo J. (2019). Prevalence of *Cryptosporidium*, *Giardia*, *Blastocystis*, and *trichomonads* in domestic cats in East China. J. Vet. Med. Sci..

[27] Li J., Dan X., Zhu K., Li N., Guo Y., Zheng Z., Feng Y., Xiao L. (2019). Genetic characterization of *Cryptosporidium* spp. and *Giardia duodenalis* in dogs and cats in Guangdong, China. Parasites Vectors.

[28] Xu H., Jin Y., Wu W., Li P., Wang L., Li N., Feng Y., Xiao L. (2016). Genotypes of *Cryptosporidium* spp., *Enterocytozoon bieneusi* and *Giardia duodenalis* in dogs and cats in Shanghai, China. Parasites Vectors.

[29] Xiao L., Morgan U.M., Limor J., Escalante A., Arrowood M., Shulaw W., Thompson R.C., Fayer R., Lal A.A. (1999). Genetic diversity within *Cryptosporidium parvum* and related *Cryptosporidium* species. Appl. Environ. Microbiol..

